# Building causal models in pain research: the case of executive functioning and transitions in pain states

**DOI:** 10.1097/j.pain.0000000000003833

**Published:** 2025-10-22

**Authors:** Annick L. De Paepe, Anna Gibby, Laura Oporto-Lisboa, Beate Ehrhardt, Matthew Nunes, Emma Fisher, Edmund Keogh, Christopher Eccleston, Charlotte Woolley, John McBeth, Geert Crombez

**Affiliations:** a Faculty of Psychology and Educational Sciences, Department of Experimental Clinical and Health Psychology, Ghent University, Ghent, Belgium; b Centre for Pain Research, University of Bath, Bath, United Kingdom; c Institute for Mathematical Innovation, University of Bath, Bath, United Kingdom; d Department of Mathematical Sciences, University of Bath, Bath, United Kingdom; e Department of Psychology, The University of Helsinki, Helsinki, Finland; f Faculty of Biology, Medicine and Health, School of Biological Sciences, University of Manchester, Manchester, United Kingdom; g Faculty of Medicine, School of Primary Care, Population Sciences and Medical Education, University of Southampton, Southampton, United Kingdom

**Keywords:** Pain, Causality, Executive functioning, Directed acyclic graphs

## Abstract

Pain states fluctuate over time and across situations. Similarly, there is variation in risk and protective factors and how they impact on these pain-related transitions. We are interested in whether such variations are more than random, and whether they can be accounted for by observed variables. The availability of large longitudinal datasets, such as UK Biobank (https://www.ukbiobank.ac.uk/), offers a unique opportunity to study these variations at scale. However, such datasets bring a high risk of bias (eg, confounding) and danger of over-interpretation. It is therefore important to be transparent about our causal thinking. Directed acyclic graphs (DAGs) are graphical representations of the hypothesized causal relationships between variables. They are used to identify the smallest set of variables that need to be adjusted to remove confounding bias in estimating the causal effect of an exposure on an outcome. However, use of DAGs in pain research is not common, despite their potential to guide study design and data analysis. In this article, we present a workflow for building a DAG using domain knowledge from 3 different sources: researchers (theory-based), people with lived experience (person-based), and the literature (evidence-based). We created a DAG for the putative effect of executive function on the maintenance of chronic high-impact pain. The resulting DAG provides a valuable framework for guiding future research on the role of executive functioning in pain, and it underscores the broader potential of using DAGs to improve causal inference in pain research.

## Abstract 2

Supplemental Digital Content is Available in the Text.

We present a procedure for developing a directed acyclic graph for the role of executive functioning on transitions in pain states.

## 1. Introduction

Chronic pain, as well as its impact, can differ between individuals and over time and is influenced by a myriad of biological, psychological, and social factors.^14,36^ An important scientific and clinical question is which factors influence an individual's transition from one pain state to another, or indeed result in the lack of transition.^12,31^ This is a challenging question to answer because of the inability to experimentally manipulate many relevant factors. Instead, we rely on natural (uncontrolled) variations that occur between and within individuals, and study how these variations differentially impact transitions in chronic pain. The role of confounding factors is a well-known problem in observational and longitudinal studies.^18^ Many researchers argue that it is impossible to infer causality from such nonexperimental designs and have avoided causal thinking. However, some authors have highlighted the dangers inherent in the avoidance of causal thinking, including the risk of bias and misinterpretation of effects.^6,8,19,54,55^ Instead, they argue for an explicit and transparent thinking about causality to avoid these mistakes.

It is important to acknowledge that causality cannot be inferred from data alone; it also requires domain knowledge. Exactly for that reason, Judea Pearl provocatively states, “Data are profoundly dumb.”^41^ For example, longitudinal data might reveal that people with poorer cognitive, or more specifically executive functioning are more likely to develop chronic pain, but it could be that those with poorer executive functioning are simply older and would have developed chronic pain regardless of their cognitive abilities. To help reinstate causal thinking, directed acyclic graphs (DAGs) have been promoted as a tool to represent the causal assumptions about the relationships between variables in a graphical model.^11,40,45,50^ A DAG thereby prompts researchers to engage in a constructive discussion about causal relationships. It helps identify the smallest set of variables needed to be adjusted to remove bias in estimating the causal effect of an exposure on an outcome (“minimal sufficient adjustment set”).

Despite their adoption in many scientific fields, there are few examples of DAGs in pain research.^30,46,51,56^ One barrier to adopting DAGs is the lack of knowledge about their use and construction.^4^ Although there is much guidance on the mathematical underpinnings of causal diagrams, few articles provide practical guidance on how to actually construct a DAG.^43^ Furthermore, when DAGs have been used, it is common for researchers not to report their method, and whether or how the DAG guided the analysis.^50^

In this article, we provide a workflow through a detailed example, complete with explanations, on how to build a DAG based on domain knowledge obtained from the empirical studies (evidence-based), researchers (theory-based), and individuals with lived experience (person-based). Our use case is the putative effect of executive function on the transitions between chronic pain states.

## 2. Methods

### 2.1. Basic directed acyclic graphs terminology

Directed acyclic graphs are graphical representations of (hypothesized) causal relationships between variables. They inform us which variables we should (not) adjust for to obtain an unbiased estimate of the causal effect of the exposure on the outcome. Adjusting for a variable can be done in several ways either statistically (eg, by adding the variable to a regression model) or by design (eg, stratification).

**Figure 1.**
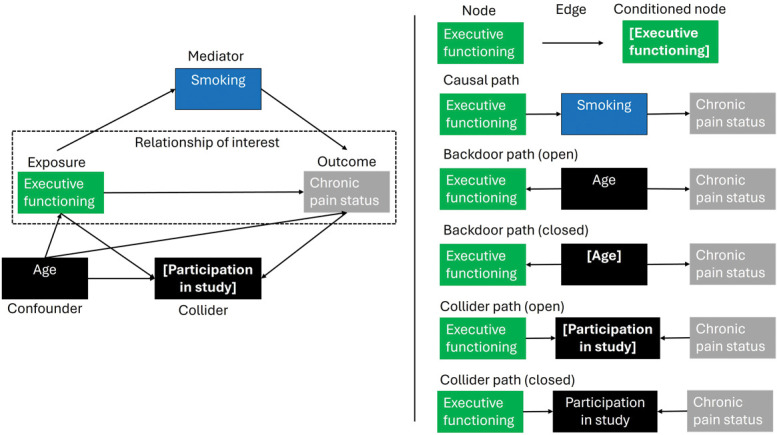
Illustration of the main components of a DAG and the most common types of paths for a hypothetical cross-sectional study in which we want to investigate the influence of executive functioning on chronic pain status (adapted from Tennant et al., 2021). This DAG has been visually arranged so that all edges flow from left to right. To investigate the average causal effect of executive functioning on chronic pain status (relationship of interest), one would have to close the backdoor path through age by controlling for this variable (eg, by adding it to your regression model). Smoking, on the other hand, is a mediator, and when interested in the total effect (or average causal effect), one should not control for this variable. As we can only observe data from people who participated in this hypothetical study and participation in the study is, according to this DAG, influenced by both executive functioning and chronic pain status, there is a noncausal path from executive functioning over participation in the study (collider) to chronic pain status that remains open and cannot be blocked. This DAG thus shows that under these assumptions, the causal effect of executive functioning on chronic pain status is not identified.

Figure 1 shows a simple DAG, in which “executive functioning,” a particular form of cognitive functioning, is the exposure and “chronic pain status” is the outcome. In a DAG, the variables are represented by *nodes.* The arrows between the nodes are called *edges* and represent a causal effect of one variable on another variable. A DAG has 2 key characteristics. First, all edges are *directed*. Hence, the direction of the causal effect is always indicated. Second, DAGs are *acyclic*, indicating that variables cannot cause themselves at the same or an earlier time point.

A *path* exists between 2 variables if they are connected by 1 or more edges (regardless of the arrow direction). *Directed or causal paths* are paths in which all arrows point in the same direction. Here, the paths from “executive functioning” to “chronic pain status” as well as the path from “executive functioning” over “smoking” to “chronic pain status” are directed. Backdoor paths between the exposure and the outcome are paths with an arrow pointing onto the exposure. For example, the path from “executive functioning” to “age” to “chronic pain status” is a backdoor path. Backdoor paths linking the exposure to the outcome are undirected or noncausal. Paths can contain a *collider*: a node that has 2 edges pointing toward it. Here, “participation in study” is a collider. These paths are also noncausal.

Paths can be *open* or *blocked*. In the example, the path “executive functioning”—“age”—“chronic pain status” is open. Any association found between executive functioning and chronic pain status can be caused by age, as age influences both executive functioning and chronic pain status. Age is called a *confounder*. Adjusting for a confounding variable blocks the path, eliminating the statistical association along the path. Adjusting for age in our example (eg, adding age as a covariate in a regression model) would eliminate the confounding bias. In the example, the path from “executive functioning” over “smoking” to “chronic pain status” is also open. However, smoking is not a confounder, but a *mediator* because it lies on the path from executive functioning to chronic pain status. If we are interested in the total effect of executive functioning on chronic pain status, this path should not be blocked or closed. If “smoking” were controlled for, part of the effect of interest would be partialled out. Paths that include a collider are blocked by the collider. In the example, the path “executive functioning”—“participation in study”—“chronic pain status” does not transmit association between “executive functioning” and “chronic pain status” unless we control for “participation in study.” Our sample is restricted to people who participated in the study, which is equivalent to controlling for “participation in study.” In turn, the path is opened, introducing a spurious (noncausal) association between executive functioning and chronic pain status.

It is crucial to add all relevant causes (ie, variables that influence either or both the exposure and the outcome) to a DAG and to control for the confounders to allow causal inference. At the same time researchers should be careful not to adjust for colliders or mediators, as this will introduce bias.

### 2.2. Constructing directed acyclic graphs

Directed acyclic graphs are constructed based on domain-relevant knowledge. Domain knowledge in this example was obtained from 3 different sources: (1) the empirical literature so that the DAG is informed by the results of available empirical studies (evidence-based), (2) the expert knowledge of a group of researchers on the topic (theory-based), and (3) the expert knowledge of a group of individuals with lived experience of chronic pain (person-based). A separate DAG is constructed for every source of information (ie, three separate DAGs). To retrieve and reconcile information from the different sources of domain knowledge we followed 4 phases based upon Rodrigues and colleagues^44^ and complemented by the steps of DAG development outlined by Poppe et al.^43^: (1) brainstorming phase: gather information from all individuals in the workshop separately; (2) refinement phase: reach consensus amongst the individuals in the workshop; (3) exposition phase: obtain feedback from the researcher group on the DAG developed by the group of individuals with lived experience of pain and the DAG developed based on the literature; and (4) reconciliation phase: combine features of all DAGs into a final DAG.

**Figure 2.**
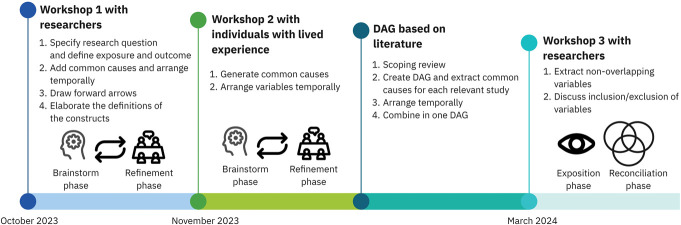
An overview of the different phases of the study.

The brainstorming and refinement phases were conducted by the researcher group and the group of individuals with lived experience of pain in separate workshops. Therefore, in the description below, these 2 phases are taken together. Two separate workshops were organised for researchers and the group of individuals with lived experience of pain to ensure that everyone felt free to communicate their ideas. A third follow-up workshop was organised for the researcher group in which the exposition and reconciliation phase was done. An overview of the different phases followed during the study are given in Figure 2.

This DAG was developed as part of the UKRI-funded programme of work, which focuses on individual and interpersonal influences on pain behaviour (Advanced Pain Discovery Platform, https://apdp.community/), and in part makes use of existing datasets, in this case the UK Biobank (https://www.ukbiobank.ac.uk/). The availability of factors measured by the UK Biobank has strongly influenced the specification of our research question and the definition of our exposure and outcome (see step 1 in section 3.1). However, we adopted a broader view, beyond the variables measured in the UK Biobank, when considering potential confounders or the relationships between variables. This was done for 2 reasons. First, it is important to consider all potential confounders in a DAG, even if these were not measured in the available dataset. Unmeasured variables can also be represented in a DAG and several methods have been suggested to deal with unmeasured confounders.^2^ Second, our aim is to provide a DAG with a more general interpretability aiding those investigating related research questions.

### 2.3. Research question of interest

Our research question was as follows: What effect, if any, do cognitive factors have on the transition between pain states, specifically the development, maintenance, and resolution of high-impact chronic pain.^12^ We decided to focus on either prospective memory or executive functioning as important cognitive factors because there is evidence to suggest their potential to predict chronic pain, and both were measured at multiple time points within the UK Biobank.^3^ Most existing studies on cognitive factors and pain are cross-sectional and have rather used a predictive than a causal framework approach.^23^

The decision to focus on prospective memory or executive functioning, as well as on the specific pain state transitions, was made during the first workshop with the researchers (step 1). During this workshop, we also discussed the exact definitions of these concepts. Specifying the research question and precisely defining all concepts is a crucial step that is often overlooked. By explicitly including this step in the DAG development process, we emphasize its importance.

### 2.4. Workshop 1 with researchers

#### 2.4.1. Participants

Eight researchers took part in a DAG development workshop. The group of researchers consisted of people with domain knowledge (A.D.P., G.C., C.E., E.F., E.K., A.G.) and people with a strong background in statistics, mathematics, and methodology (A.D.P., M.N., L.O.L.). Details with regard to the affiliation and background of the researchers can be found in the supplemental digital content (see SI-Table 1, http://links.lww.com/PAIN/C402). All researchers were part of the “High-Impact Chronic Pain and UK Biobank” (CHIPP) project, which aims to increase our understanding of long-term transitions of chronic pain and the causal relationships with predisposing factors (such as cognitive factors).

#### 2.4.2. Procedure

Before the first workshop, researchers received some training and reading material to become acquainted with the rationale and basic terminology of DAGS. An in-person half-day introduction to causal DAGs was given by A.D.P. to the researchers in January 2023. This was followed by a 1-hour online “refresher” course in August 2023. Moreover, several introductory articles on DAGs were shared with the researcher group. We considered it unnecessary for domain experts to fully understand the mathematical graph theory underpinning DAGs, but the rationale of their use in research was deemed necessary to be able to contribute in a meaningful way to the development of the DAG.

The first workshop was held in person in October 2023 over 2 half days. The purpose was to create a DAG for 1 specific research question. Three steps were followed to construct the DAGs as outlined by a recent scoping review on guidelines for DAG development^43^: (1) specify the research question and define the exposure and outcome; (2) add common causes and arrange temporally; (3) draw forward/directed arrows indicating the assumed causal relationship between 2 variables. On the first day, we focused on steps 1 and 2. The second day was devoted to finishing steps 2 and 3.

##### 2.4.2.1. Step 1. Specify the research question and define exposure and outcome

In the brainstorm phase, all researchers were asked to think individually about 2 questions: (1) “If we had all the data that we wanted, what is/are the most interesting research question(s) that you would like to answer?” and (2) “Given the data that we have, which research questions are feasible to answer?” Researchers were asked to type their thoughts on virtual post-it notes that they could add to a shared MIRO board (https://miro.com/nl/).^37^

In the refinement phase, the individual responses were discussed in a group, and a consensus was reached on the specific research question of interest that would be focused upon. We subsequently discussed how to define the exposure and the outcome within the context of our research question.

##### 2.4.2.2. Step 2. Add common causes and arrange temporally

In the brainstorm phase, researchers thought individually about possible common causes of the exposure and the outcome (also called “confounders”). They were asked to order these common causes temporally, from left (variables that were fixed early in the life of the participants) to right (variables that are time-variant or were only fixed more recently). Researchers were asked to type their thoughts on virtual post-its and add these to the shared MIRO board.

In the refinement phase, a group discussion evolved around the common causes and the mediators of our research question of interest. First, variables that were essentially the same but were formulated differently were merged together. For example, “early pain experiences” and “pain exposure during early years” were combined to “early pain experiences.” Second, we thought about which groups of variables occurred at similar time points and had similar relationships to the other variables that were specified in the DAG. These variables were grouped together in what is sometimes called “super-nodes.”^50^ For example, age, socioeconomic status, education, profession, country of origin, and first language were taken together in the super-node “Demographic factors.” This improves the clarity of the DAG and manages its complexity. Relationships between variables within the same super-node can be left unspecified, as these are not relevant to the causal effect of interest. Note that the use of super-nodes is only intended to enhance readability and manage complexity. This does not imply that the individual variables within a super-node are interchangeable or redundant. Each variable remains conceptually and analytically important and should be included separately in statistical models. Third, we discussed the most important mediators between the exposure and the outcome. Step 3. Draw forward arrows (edges).

All the forward arrows (ie, arrows going towards variables to the right in the DAG) were added to the DAG, and a group discussion evolved around which arrows to leave out of the DAG. This is sometimes referred to as a “dismantling approach” in which you start from a full, saturated DAG (depicting all edges) and systematically remove unplausible edges. This stands in contrast to an “additive approach,” in which edges are added sequentially.^43^ If there was uncertainty about whether an arrow should be removed, we decided to keep the arrow in the DAG, as removing an arrow is a stronger assumption of no association than keeping the arrow in Ref. 50.

### 2.5. Workshop 2 with individuals with lived experience

#### 2.5.1. Participants

Individuals with lived experience were selected from the CHIPP patient advisory group. This is a group of 25 individuals with lived experience of chronic pain who advise on the different work packages of the project. Five to 10 members of PPIE communities were invited through each of the 4 collaborating institutions (Keele University, University of Bath, University of Manchester, and University of Aberdeen). The final 25 group members were selected based on age, situation (employed/retired/carers/students), and other factors such as living in a deprived area/having a lower income/identifying as an ethnic minority to ensure diverse representation. In selecting a panel for workshop 2, all advisory group members were invited to participate. Owing to a limited uptake, we were not able to purposively select participants to ensure diversity. However, we did have variation in the background of group members who volunteered to engage.

Seven people with lived experience of chronic pain (6 women, 1 man) took part in a workshop that was facilitated by 2 researchers (A.G. and A.D.P.). Six of the participants had a formal diagnosis of 1 or more pain conditions. The conditions reported were fibromyalgia (N = 4), osteoarthritis (N = 2), neuropathy (N = 1), or another unspecified condition (N = 2). Pain was reported in the back, muscles/tendons/joints, head, or all over. Participants ranged from 25 to 70 years old—5 individuals reported being from a lower income, and 2 reported belonging to an ethnic minority group. Employment status included working (N = 3), primary carer (N = 2), student (N = 1), and retired (N = 1). The Guidance for Reporting Involvement of Patients and the Public Short Form (GRIPP2-SF) reporting checklist^49^ was completed and can be found in supplemental digital content (see SI-Table 2, http://links.lww.com/PAIN/C402).

#### 2.5.2. Procedure

A 2-hour online meeting was held in November 2023. During this meeting, a short introduction was given about the aims of the research project in nontechnical terms by 1 of the facilitators. The independent and dependent variables were introduced as “thinking patterns/cognitive factors” and “chronic pain experience” as a lay substitute for cognitive factors and the impact of chronic pain, respectively, and they were briefly explained. Also, “transitions of impact in pain” were explained briefly, and we explained that within this study, we focused specifically upon the maintenance of high-impact chronic pain. Finally, we introduced the main question that we would focus upon during the workshop as follows: “We predict that thinking ability can cause chronic pain status over time. We need help thinking about what other factors might impact this relationship.” An example was given to clarify (depression as a common cause of thinking ability and chronic pain status).

During the workshop with the group of individuals with lived experience of pain, we planned to focus on “step 2” of the researcher workshop, namely “add common causes and arrange temporally.” However, because of the limited time available, we were not able to include input from individuals with lived experience on the temporal ordering of the factors. This step was then done by the facilitators of the workshop (A.G. and A.D.P.) and was subsequently discussed with the co-authors of this article.

In the brainstorm phase, the group of individuals with lived experience of pain was asked to generate any “thinking factors” they believed influenced their “chronic pain experience.” They were asked to type down their thoughts on virtual post-its and add them to the shared MIRO board.

During the refinement phase, we asked the group of individuals with lived experience of pain to expand on factors that were less clear to the facilitators. Next, the facilitators grouped the factors into “super-nodes,” similar to the researcher group. Finally, each one in the group of individuals with lived experience of pain was asked to outline the 3 factors that they felt were most important in affecting their pain experience.

### 2.6. Directed acyclic graphs based on literature

Next, we identified factors in the existing empirical studies on this topic. Articles were considered for inclusion based on the following criteria:

(1) The study was a prospective, longitudinal design
(2) Pain status was measured at both baseline and follow-up
(3) Executive function was explored as a predictor of pain
(4) Participants were adults (18 years or older)

Studies that met these inclusion criteria were identified in several ways. First, based on the 734 included articles for another review (search conducted in March 2022) with similar but broader inclusion criteria, from colleagues at the Advanced Pain Discovery Platform (ADPD) Consortium to Research Individual, Interpersonal and Social Influences in Pain (CRIISP, https://criisp.uk/). These articles were scanned to identify those that matched our more focused inclusion criteria. Five studies were identified in this way. Second, a brief search of the literature was done in PubMed and Google Scholar using key terms relevant to the inclusion criteria. The terms used were “executive function and pain,” “executive function and chronic pain,” “cognitive factors and chronic pain,” and “Trail Making Task and Pain,” as these were most relevant to the main research question. This search was limited in terms of timeframe (from 2021 to present, as this period was not included in the review conducted by the CRIISP consortium) and in scope (articles had to be longitudinal and include the impact of executive function on pain outcomes to identify confounders considered for both). Six articles were identified based on this search, but none of them met the inclusion criteria upon closer inspection (different exposure, no prospective longitudinal design). As a final check, to ensure that no important articles were missed, the researcher group involved in workshop 1 was asked to identify any articles that may have been missed. No additional articles were identified based on input from the researcher group.

For each relevant study, the variables that were controlled in these studies were extracted by A.G. and A.D.P., and a separate DAG was created for each study. Subsequently, all variables identified were temporally ordered and integrated into 1 overarching DAG.

### 2.7. Workshop 3 with researchers (follow-up)

#### 2.7.1. Participants

Seven of the 8 researchers who participated in the DAG development workshop took part in a follow-up online workshop for the exposition and reconciliation phase in March 2024.

#### 2.7.2. Procedure

The workshop focused on the exposition and reconciliation phase. The background and aims of the study and a summary of the different steps that had already been taken in the DAG development process were presented. Before the workshop, A.G. and A.D.P. had compared the 3 DAGs and extracted those factors that were identified by the group of individuals with lived experience of pain and/or the literature but were not present in the researcher DAG. During the workshop, the research team was presented with these new factors and the corresponding reasoning of the group of individuals with lived experience of pain or the literature (exposition). The team discussed whether to include these factors either as common causes or as mediators (reconciliation).

## 3. Results

### 3.1. Workshop 1 with researchers

#### 3.1.1. Step 1: Specify research questions and define the exposure and the outcome

Brainstorming resulted in a broad and varied set of research questions, which can be found in supplemental digital content (see SI-Fig. 1, http://links.lww.com/PAIN/C402). In the refinement phase, a consensus was reached among the researchers to focus upon “Executive functioning” as exposure and on the “Maintenance of high-impact chronic pain” as outcome. Researchers agreed that there were sufficient arguments that executive functioning was a potentially relevant factor for chronic pain outcomes, but the causal effect of executive functioning on chronic pain outcomes was yet to be established.^3,48^ Furthermore, the researchers were particularly interested in the maintenance of high-impact chronic pain and whether poor executive functioning could lead people to become “stuck” in their pain state.^5,12^

Subsequently, we discussed how best to conceptualize our exposure and outcome.

##### 3.1.1.1. Exposure

Executive functioning involves higher-order cognitive strategies that control stimulus information and subsequent behaviour, identified by tests of inhibition, shifting, and working memory updating.^34^ Inhibition involves suppression of prepotent responses (ie, response inhibition) and selectively attending to motivationally relevant stimuli, while not attending to irrelevant stimuli. Shifting involves the ability to switch between mental sets and rules, and updating involves monitoring information in working memory with addition, suppression, and deletion. Within the UK Biobank, executive functioning was measured using 2 different tasks: “Trail Making Test A and B” and “Tower of London.” Different components of executive functioning are measured by the 2 tasks (Table 1). We decided to focus on the Trail Making Test parts A and B as independent variables because this test measures components of executive functioning that we considered most relevant in the context of the maintenance of chronic high-impact pain. Moreover, we decided to work with the difference score between part B and part A of the Trail Making Test, as this has been shown to provide a relatively pure indicator of executive control abilities, predominantly measuring switch cost.^47^ Consequently, executive functioning was defined here as the ability to maintain attention, process information quickly, and switch between tasks efficiently.

**Table 1:** Details of the 2 tasks measuring executive functioning included in the UK Biobank.

	Components of executive function measured*	Outcome
Trail Making Test part A and part B	Cognitive control Serial processing Visual search	Time (in deciseconds) to complete part A and part B
Tower of London (imagine)	Strategy Speed of planning	Number of items answered correctly in 3 minutes

* Components of executive functioning are based on the cognitive atlas (https://www.cognitiveatlas.org/).

##### 3.1.1.2. Outcome

We were interested in the maintenance of high-impact chronic pain. To investigate this, participants who had high-impact chronic pain at baseline and follow-up were compared with participants who had high-impact chronic pain at baseline, but whose functioning improved at follow-up to chronic pain with a low impact. Definitions of chronic pain, low- and high-impact chronic pain, and the transitions in pain states are presented in Table 2.

**Table 2:** Definitions of chronic pain, high- and low-impact pain, and transitions in pain states taken from Ref. 10.

	Definition
Chronic pain	Pain that has lasted for 3 mo or more
High-impact chronic pain	Chronic pain with a high impact on major self-care, occupational, or social activity restrictions
Low-impact chronic pain	Chronic pain with a low impact on major self-care, occupational, or social functioning
Onset of chronic pain	Change from no pain to chronic pain with a low or high impact
Maintenance of chronic pain	No change in chronic pain state
Worsening of chronic pain	Change from chronic pain with a low impact to a high impact
Improvement of chronic pain	Change from chronic pain with a high impact to a low impact
Recovery of chronic pain	Change from chronic pain with a low impact to no pain

##### 3.1.1.3. Measurement time

UK Biobank collected data at baseline (2006-2010) and at 3 follow-ups (2013, 2014, 2019). Of these, executive functioning was not measured at baseline (2006-2010) but was only measured at follow-ups. However, there is evidence of inconsistent validity of outcomes for the cognitive battery in the 2013 follow-up,^34^ as well as a limited sample size of approximately 20,000 participants (which represents only 4% of the total sample). We therefore decided to focus on the measurement starting in 2014 as “Baseline measurement” or T1. The next measurement period in 2019 was taken as a “follow-up measurement” or T2. It is important to note that although these measurement periods started in 2014 and 2019, respectively, not every participant completed the assessments in those years. For the cohort used in the analyses, we will ensure there is at least a 6-month interval between the 2 measurement points. This approach was chosen because we believe a minimum of 6 months is necessary for any changes in the impact status of chronic pain to become evident.

##### 3.1.1.4. Research question

To correctly specify a research question, it is important to specify the theoretical causal estimand.^33^ This is a description of the exact intervention effect a study aims to quantify without direct reference to the data at hand and the statistical analysis technique one aims to use. The theoretical estimand consists of 2 main components: a contrast of potential outcomes and the target population. By specifying the estimand information is provided on the following aspects: (1) causal effect of interest: the desired causal effect of the exposure on the outcome; (2) exposure of interest: the values or levels of the exposure at which one expects a change in the outcome (eg, clearly defining “good” vs “poor” executive control); (3) outcome of interest: it is important to choose an appropriate time window to study the effect of the exposure on the outcome. One has to ensure that the outcome is separated in time from the exposure, because a cause should precede the effect. If the exposure is highly variable, it may be necessary to choose a smaller time window. Alternately, if it takes time before the exposure can have an effect on the outcome, then it is necessary to choose a larger time window; and (4) target population: a DAG is only valid within a specific population or context. If one wants to investigate the same research question in a different population or context (eg, a different country), one has to reassess whether all relationships specified within the DAG are still valid.

Our research question is: What is the average causal effect of low executive functioning (a score below the population norm on the Trail Making Test) compared with normal or higher executive functioning (a score equal to or higher than the population norm) at time 1 on the maintenance of high-impact chronic pain at time 2 (at least 6 months later) in those with high-impact chronic pain at time 1 (Table 3).

**Table 3:** Different components of the research question.

Causal effect of interest	Average causal effect: the average change in the outcome that can be attributed to a difference in the exposure.
Exposure	A score below the population norm on the Trail Making Test (ie, poor executive functioning) as opposed to a score equal to or higher than the population norm (ie, normal executive functioning) at baseline
Outcome	The maintenance of high-impact chronic pain at time 2
Time difference between baseline and follow-up	At least 6 mo (T1-T2)
Population	Respondents of the UK Biobank who have chronic pain of high impact at time 1

#### 3.1.2. Step 2: Add common causes (or confounders) and arrange temporally

The results of the brainstorming phase are presented in supplemental digital content (see SI-Figure 2, http://links.lww.com/PAIN/C402). Researchers spontaneously brought up some of the mediating pathways between the exposure and the outcome. Because we are interested in the average causal effect, controlling for mediators could lead to an underestimation of the influence of executive functioning on the maintenance of high-impact chronic pain. Therefore, we decided to also think about the most important mechanisms by which executive functioning could influence the maintenance of high-impact chronic pain, to make sure that we do not mistakenly take these variables into account as possible confounders.

**Figure 3.**
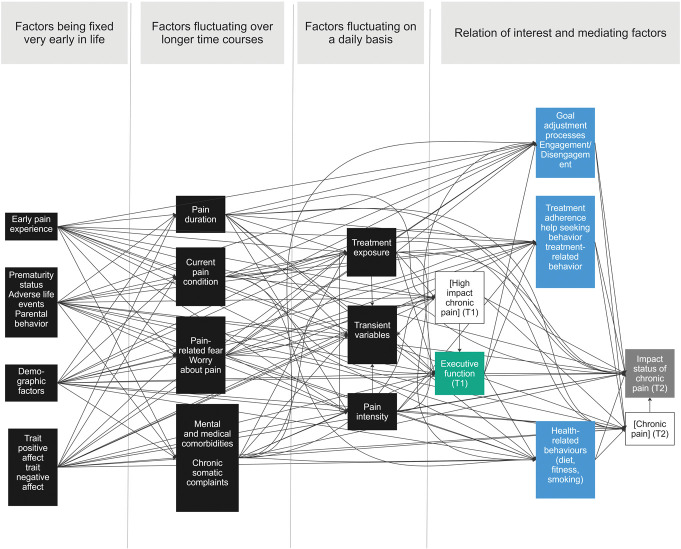
Final DAG for the researcher group at the end of step 3. Green box = exposure; gray box = outcome; blue box = mediator; black box = common cause; white box in square brackets: variables that are fixed at a given value (ie, only people with high-impact chronic pain were included at T1, and only people with chronic pain at T2).

The result of the refinement phase can be found in supplemental digital content (see SI-Figure 3, http://links.lww.com/PAIN/C402).

A consensus was reached about most of the common causes and mediators. There was uncertainty about whether to include “Lifestyle factors” as a mediator or a confounder. We decided to define it more precisely as health-related behaviours (diet, physical activity, treatment adherence, smoking) and to add it as a mediator. Nevertheless, we decided to conduct a sensitivity analysis later, looking at the difference in the average causal effect when considering it as a confounder as opposed to a mediator.

#### 3.1.3. Step 3: Draw forward arrows

The results of this phase are shown in supplemental digital content (see SI-Table 3, http://links.lww.com/PAIN/C402).

We decided to leave the genetic and environmental factors out of the DAG, because we considered the effect of these variables on executive functioning and impact status of chronic pain at follow-up to be mediated by more proximal variables, such as comorbidities or trait positive and negative affect. Controlling for those more proximal variables can already block the path, making it unnecessary to also control for genetic factors.

#### 3.1.4. Elaborating on the definitions of the constructs included in the directed acyclic graphs

To be sure that all constructs entered in the DAG were in the appropriate place, it is important to be explicit about the definitions of the constructs. Therefore, after the 2-day DAG development workshop, clear definitions were added for the constructs present in supplemental digital content (see SI-Figure 3, http://links.lww.com/PAIN/C402). Definitions were as far as possible based on existing definitions and were discussed within the group of researchers until a consensus was reached. By clearly defining all the concepts within the DAG, we became aware of some overlap between the constructs that were present within the DAG (eg, pain anxiety and pain-related fear). Moreover, we decided to omit some factors at this point, because when reconsidering them, we judged that a direct relationship between the construct and either the independent or dependent variable was unlikely (eg, after consulting with experts on pain-related stigma, stigma was judged unlikely to have a strong association with executive functioning). An overview of the changes made at this point is provided in supplemental digital content (see SI-Table 4, http://links.lww.com/PAIN/C402). The final definitions of the included constructs can also be found in supplemental digital content (see SI-Table 5, http://links.lww.com/PAIN/C402). The resulting DAG for the researcher group is shown in Figure 3.

### 3.2. Workshop with people with lived experiences

#### 3.2.1. Step 1: Generation of common causes

**Figure 4.**
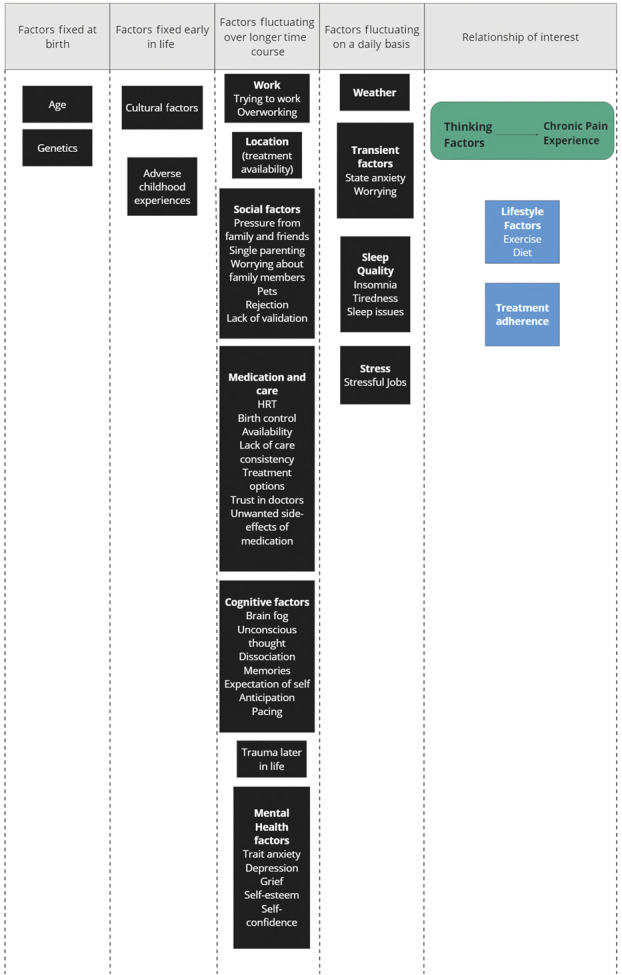
Overview of all the common causes and potential mediators that were identified for the research question of interest by the ILE group. Common causes were put in temporal order by the researchers. Green box = exposure and outcome; blue box = mediator; black box = common cause.

The 60 factors that were generated in the brainstorm phase can be found in supplemental digital content (see SI-Fig. 4, http://links.lww.com/PAIN/C402).

The grouping of the factors and their description can be found in supplemental digital content (see SI-Table 6, http://links.lww.com/PAIN/C402). The factors mentioned as most important for people with lived experience included lack of care consistency (N = 1), trauma (N = 2), invisible illness (N = 1), weather (N = 2), stress (N = 1), pacing (N = 2), mental health (N = 1), sleep (N = 3), being validated (N = 1), brain fog (N = 1), clarity (N = 1), expectation (N = 1), dissociation (N = 1), and energy (N = 1).

#### 3.2.2. Step 2: Arrange the factors temporally

After the meeting, the facilitators put the common causes identified by the group of individuals with lived experience of pain in temporal order. The results are shown in Figure 4.

### 3.3. Directed acyclic graphs based on literature

**Figure 5.**
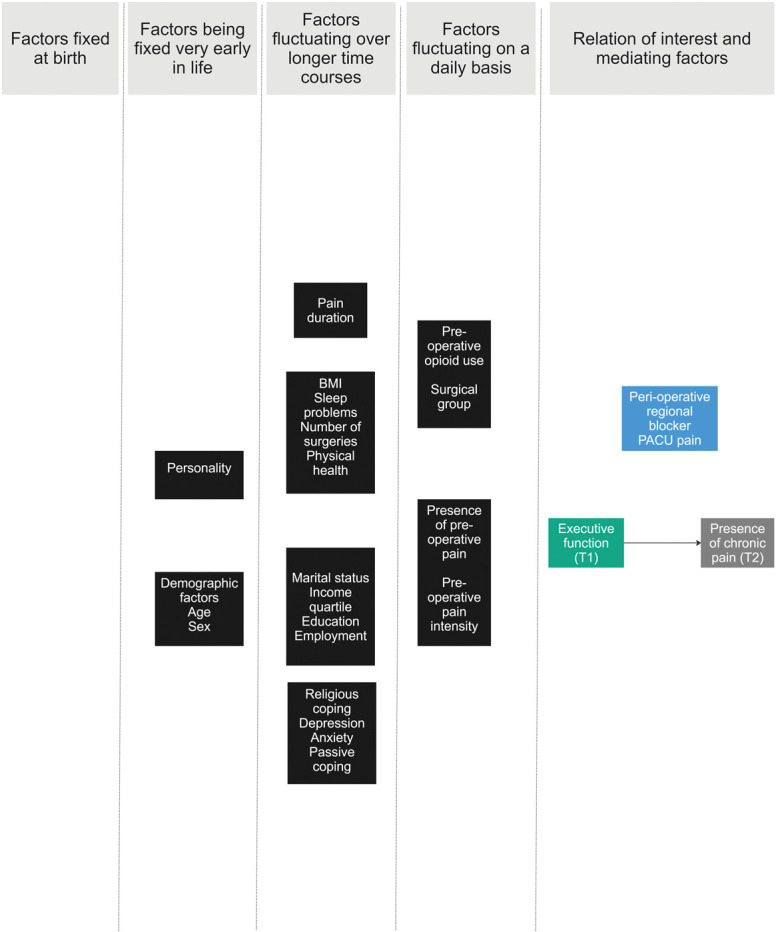
Overview of all the common causes and potential mediators that are potentially relevant for the research question in the literature. Common causes were put in temporal order by the researchers. Green box = exposure; gray box = outcome; blue box = mediator; black box = common cause.

Five articles were identified as matching the inclusion criteria.^3,15,16,38,53^ In these studies, 1 to 8 confounders were considered in the statistical analyses. None of the studies reported to have constructed a DAG to answer their research question. An overview of all the common causes and potential mediators identified for the research question based on the literature is given in Figure 5. It is important to note that all included studies focused on the development of chronic pain, whereas our research question focuses on the maintenance of chronic pain. Moreover, 4 out of the 5 studies had only pain severity and the presence of chronic pain as outcomes, and 1 study had pain interference as a secondary outcome. By contrast, our focus is on the impact of chronic pain. Details of the studies and DAGs constructed based on the information given in the articles are outlined in supplemental digital content (see SI-Table 7, http://links.lww.com/PAIN/C402).

### 3.4. Workshop 3 with researchers (exposition and reconciliation)

There was substantial overlap in the common causes suggested by the researcher group and the group of individuals with lived experience of pain. Based on the suggestions of the group of individuals with lived experience of pain, we decided to add “lack of care consistency” and “pacing” as mediators, and “medication side-effects” and “trauma later in life” as common causes. Factors that were suggested by the group of individuals with lived experience of pain that were not included in the final DAG were contextual factors (eg, treatment availability, the weather), social factors (eg, the role of pets, stigma, social support), cognitive factors (eg, memory, brain fog), and genetic factors.

Based on the DAG constructed based on the literature, it was decided to add “income quartile,” “employment,” and “profession” as more precise indicators of SES and to include “sex” under demographic factors. Other variables, such as certain coping strategies (eg, religious coping, passive coping) or personality traits, were not included.

**Figure 6.**
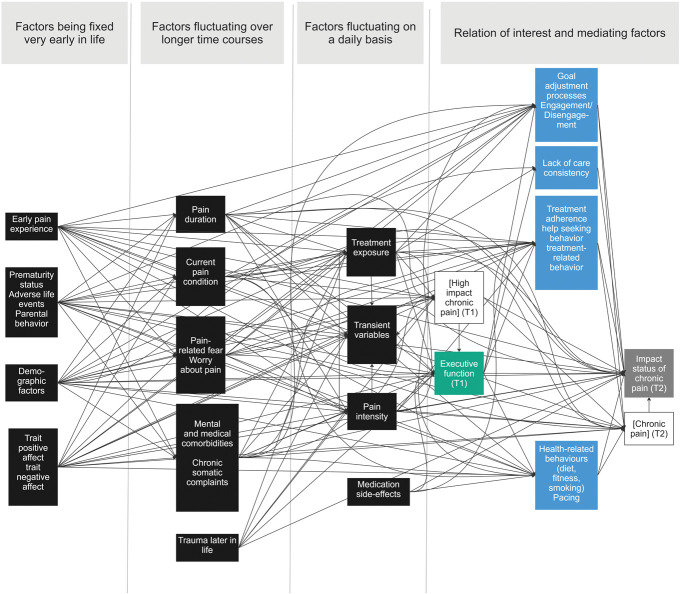
Final DAG after the exposition and reconciliation phase. Green box = exposure; gray box = outcome; blue box = mediator; black box = common cause; white box in square brackets: variables that are fixed at a given value (ie, only people with high-impact chronic pain were included at T1, and only people with chronic pain at T2).

Extensive reasoning for (not) including the variables suggested by the group of individuals with lived experience of pain and in the literature is reported in supplemental digital content (see SI-Table 8, http://links.lww.com/PAIN/C402). The final DAG is depicted in Figure 6.

## 4. Discussion

Many researchers are interested in the effects of particular variables (exposure) on pain (outcome), also in nonexperimental designs. Such causal questions are fundamentally different from predictive questions.^23^ To make causal claims from nonexperimental designs, it is quintessential to carefully consider variables that might bias the estimate of the hypothesized causal relationship.^6,18,43,50^ These considerations are based upon domain knowledge, and can be visualised in causal DAGs. There are a few examples of DAGs in pain research. Overall, there is little guidance on how to construct a DAG. In this study, we provide such guidance while constructing a DAG for the putative effect of executive functioning on the transition between chronic pain states. This example may offer an inspiring workflow that can be flexibly adapted by other researchers to develop their own DAG.

We described a workflow for a research question that can only be addressed by nonexperimental research. Considerable time was spent formulating a precise research question and clearly defining the exposure and the outcome. A helpful approach is to define the causal effect as if the effect would occur in a hypothetical trial (ie, trial emulation).^10,24,25^ Such emulation includes specifying the population under study (or the eligibility criteria), the exposure (or intervention strategy), the outcome (the primary outcome), and the causal contrast of interest. Doing so ensures an adequate level of specificity of the causal contrast of interest (eg, the average treatment effect), the “active treatment” condition (eg, poor executive functioning), the comparator condition (eg, good executive functioning), its operationalisation (eg, a score above the population mean vs below the population mean on the Trail Making Test), and the time window between exposure and outcome (eg, 6 months).

In nonexperimental designs, causal questions will require adjustment for confounding variables to achieve comparability (or exchangeability) between groups with different levels of exposure. Several guidelines are available to determine whether a variable qualifies as a confounder.^52^ In this study, we followed the “common cause” approach.^17^ Preexposure factors that influence both exposure and outcome were selected and put in temporal order from 3 sources of information: theory-based (researchers), evidence-based (empirical studies), and person-based (individuals with lived experience). Each source provided some unique variables, highlighting the value of using diverse perspectives. This resulted in the identification of 18 confounders, which is far more than the usual number of confounders in empirical studies on this topic.^3,15,16,38,53^ Notwithstanding, it remains possible that some confounders were missed. The major benefit of creating DAGs is, however, not in the true representation of reality, but in its transparency and argumentation, explicitly outlining the assumptions underpinning the estimated causal effect. This aligns with the principles of open science^9^ and provides firm ground for scientific discussion. In many statistics courses, the mantra “Correlation does not imply causation” has led to avoiding causal language and thinking in the methods and results sections of articles. Nonetheless, causal language often slips into the general discussion. For example, Giusti et al.^16^ discussed executive functions “shaping” pain outcomes, implying causality despite using a predictive approach. Using a predictive approach for causal questions is a common mistake, leading to misinterpretation of results and even erroneous conclusions for practice or policy.^19,23^ It is therefore important to be aware of the distinction between a predictive or causal approach, and to be transparent about it.^20^

The “common cause” principle identifies confounders as variables that *happen prior to the exposure* and that have an influence on the outcome. This timing is crucial to avoid mistaking mediators for confounders, as adjusting for mediators may induce rather than reduce bias. A mediator explains part of the process through which an exposure affects the outcome, so adjusting for it would remove some of the effect of interest. Unfortunately, in practice, exposure and potential confounders are often measured at the same time point. While it is obvious that some variables occurred before the exposure (eg, early pain experiences), it is less clear for others (eg, physical activity). This complicates decisions on whether to classify a variable as a confounder or a mediator. During the workshops, we encouraged participants to also think about mediators, to ensure that we would not adjust for these variables. Sometimes, uncertainty remained about whether a variable was more important as a confounder or as a mediator. We kept track of this information, and propose to conduct sensitivity analyses to determine the influence of adjusting or not adjusting for these variables.^50^

In addition, confounders and mediators need to be properly defined. We were able to merge or exclude some of these factors due to content overlap or to changes in their temporal order when considering a precise definition. Being explicit and transparent about causal assumptions also requires being explicit and transparent about definitions of the included concepts. There is increasing awareness that some often-used concepts are poorly defined^8,13,42^ and efforts have been made to provide an overview of definitions of commonly used concepts within pain research and to offer guidance on how to do better.^7,26,39^

The DAG presented in this article is a conceptual or “dream” DAG, reflecting the conditions under which we would have the greatest confidence in drawing causal conclusions. To apply this in practice, it will need to be translated into an empirical DAG, a version of the “dream” diagram informed by the actual variables in the data set. Starting from a conceptual DAG helps to ensure that we think beyond the data at hand, making us more aware of potential sources of residual bias. It is important to ensure that the variables are operationalized so that they comprehensively match the definition of the concepts to minimise measurement bias.^21^ If some of the variables are not available, they can be included in the DAG as “unmeasured,” and one may consider conducting bias analyses for uncontrolled confounding.^2,35^ Also, the way in which participants were recruited can be represented in the DAG to avoid selection bias.^22,32^ For example, in the UK Biobank, there is evidence that the individuals included are more likely to be older, female, and of higher socioeconomic status than the general UK population. This could lead to spurious associations (eg, older individuals in the UK Biobank tend to be in better health). There are ways to remediate this problem, such as inverse probability weighting.^1^

Our workflow is detailed and extensive, and this may not be required for each study. However, we hope that the description helps others to adopt and adapt our workflow. Researchers may choose to begin by drafting a preliminary DAG based on their own expertise and then iteratively refine it through consultation with literature, domain experts, and individuals with lived experience. We do, however, believe that a final DAG will likely be theory-based (knowledge of researchers/clinicians), evidence-based (empirical evidence), and person-based (expertise of individuals with lived experience). Ideally, DAGs are developed before data collection to guide study design (Poppe et al., 2025). However, our comprehensive approach may be particularly valuable when working with established large-scale datasets, such as biobank data. Constructing a well-informed DAG can help researchers maximize data utility while maintaining transparency about potential bias sources. Table 4 provides a summary of our hands-on recommendations in developing a DAG.

**Table 4:** Summary of recommendations for developing a DAG based on 3 sources of information (theory-based, evidence-based, and person-based).

DAG development step	Recommendation
Consultation of experts (theory-based)	Ensure a good distribution between people with a methodological/statistical background and people with subject matter expertise relevant to the research question of interest Ensure that every researcher has a basic understanding of what DAGs are and how they are used
Consultation of people with lived experience of pain (person-based)	Ensure that you have a diverse representation that reflects the breadth of the condition under study Make sure to capture causal relationships that are true at a group-level (on average) and that are not specific to a single person
Consultation of existing empirical studies (evidence-based)	If a systematic literature review on the topic of interest already exists, start from this At the very least, screen the literature for studies with similar research questions and critically assess which variables were included as confounders, mediators, and colliders and whether it is important to include these variables in your own study Be aware that most previous studies were focused on prediction instead of causality, and significant associations identified within these studies are not necessarily causal

Our approach may be complemented by other approaches. For example, machine-learning tools might help researchers to improve confounding adjustment.^27^ These may be especially relevant when using databases that are not collected for the specific research question of interest.^57^ However, we argue that domain knowledge remains necessary in inferring causality. Including confounders without background knowledge increases the likelihood of bias.^28^

This study has some limitations. First, we incorporated the perspectives of researchers and individuals with lived experience of pain, but did not involve other perspectives (eg, clinicians). In addition, our research team was primarily composed of experts in psychology and data analysis. Second, owing to resource constraints, individuals with lived experience of pain could not participate throughout the entire development process. Third, the theory-based DAG relied on literature from similar but not identical research questions. Fourth, we mainly focused on the assumption of “no unobserved confounding.” There are other assumptions that need to be checked (see Ref. 29). Fifth, no DAG is “perfect.” A DAG's value lies in making assumptions explicit and transparent, prompting discussion and feedback. As a primarily didactic manuscript, our aim was to illustrate DAG construction rather than contribute empirical or theoretical insights into pain mechanisms. In this spirit, we acknowledge that reviewers provided valuable comments worth sharing with readers. Concerns were raised about treating socioeconomic variables—such as income and employment—as fixed. Other comments questioned the exclusion of genetic and environmental variables and the selective focus on negative affectivity, while overlooking traits like conscientiousness or openness. Our rationale was that many of these broader influences are likely mediated through more proximal variables already included in the model. However, we recognize that this approach may overlook genetic or environmental factors that exert direct effects that are not fully captured by the included variables and that broader trait dimensions could be relevant.

## Conflict of interest statement

The authors have no conflicts of interest to declare.

## Supplementary Material

## Appendix A. Supplemental digital content

Supplemental digital content associated with this article can be found online at http://links.lww.com/PAIN/C402.
