# Supplementary material for: Building causal models in pain research: the case of executive functioning and transitions in pain states
Source: Pain. 2025 Oct 22;167(2):414–27. doi: 10.1097/j.pain.0000000000003833 (PMC12794360; doi:10.1097/j.pain.0000000000003833)
Supplement: Supplementary file 1 [file jop-167-414-s001.pdf]

Supplementary information

SI-Figure 1

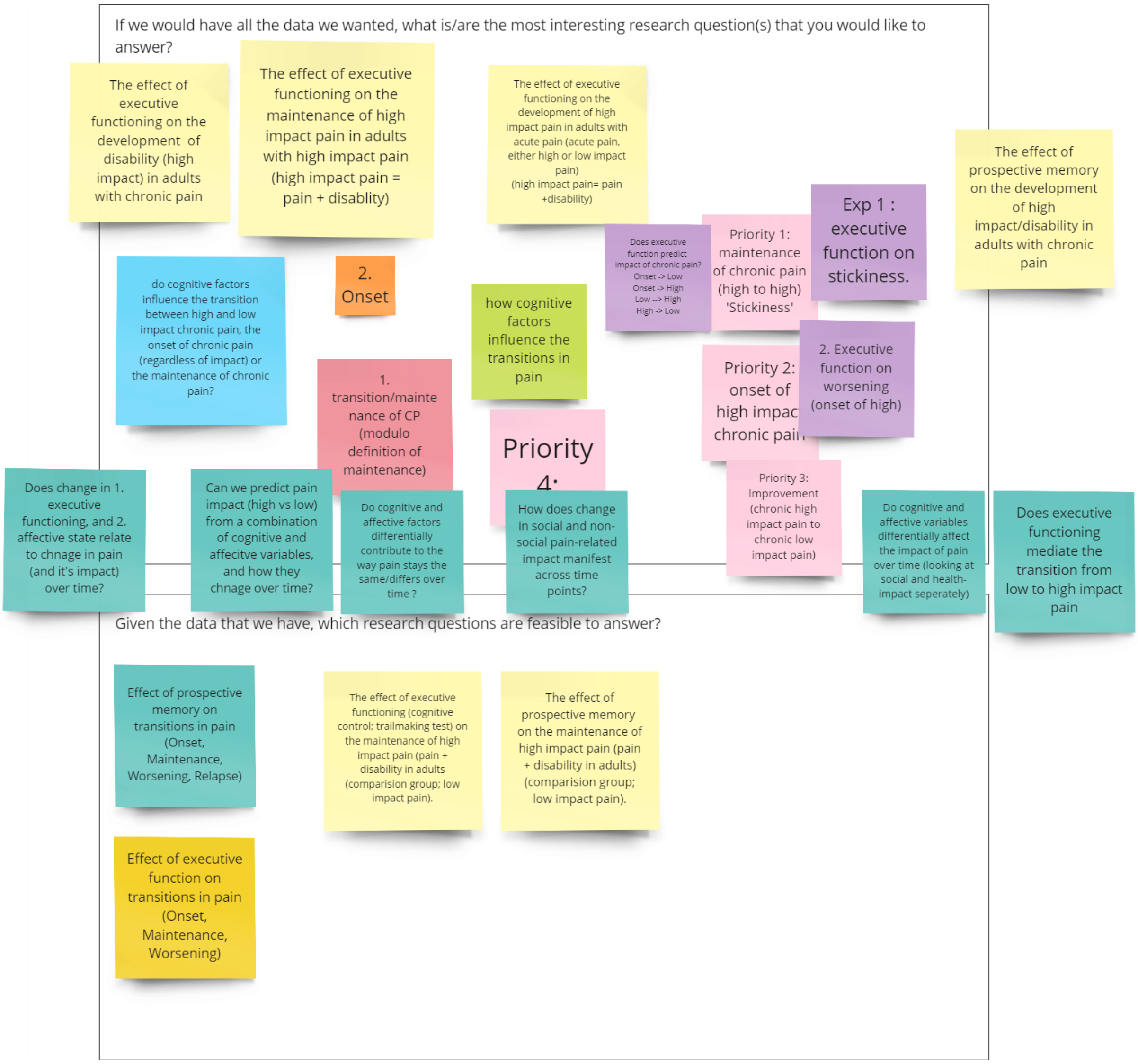

SI-Figure 1. Results of brainstorm phase with researchers on step 1 (Specify research question).

SI-Figure 2

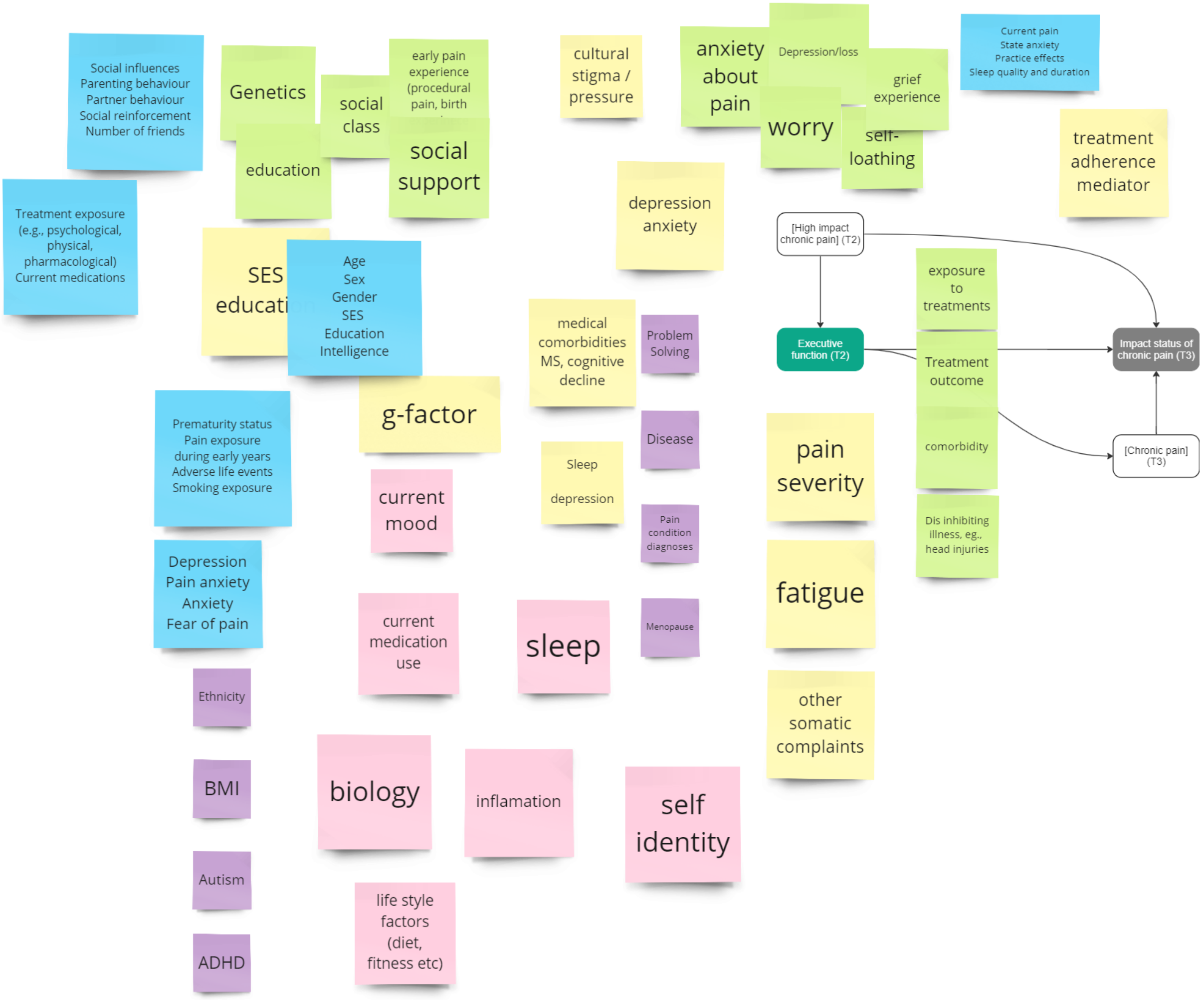

SI-Figure 2. Results of the brainstorm phase in step 2 (Add common causes and arrange temporally).

SI-Figure 3

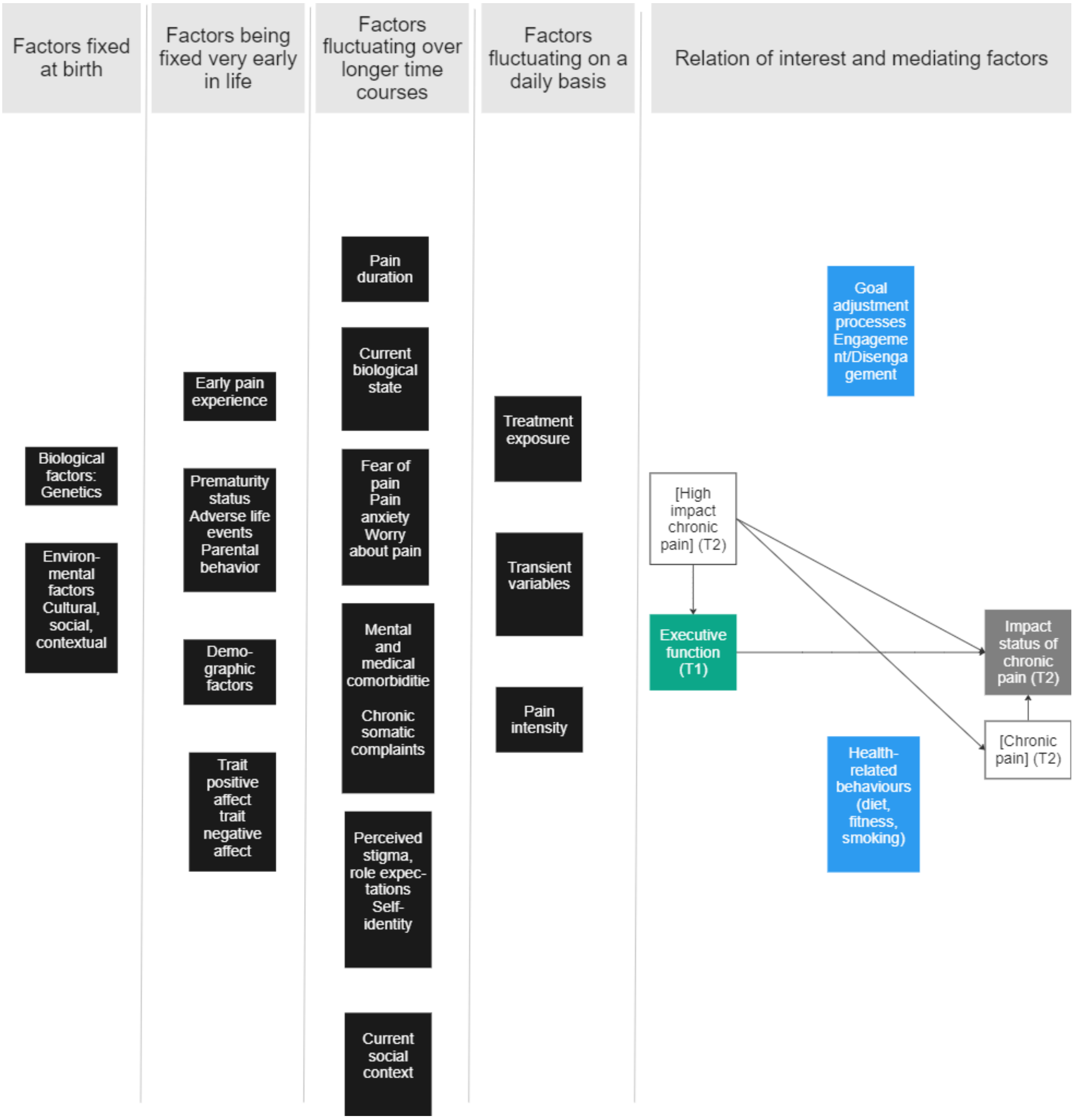

SI-Figure 3. Result of the refinement phase of step 2. Overview of all the common causes (black) and potential mediators (blue) that were identified for the research question of interest. Common causes were put in temporal order. High impact chronic pain at baseline and chronic pain at follow up are between brackets to indicate that they are controlled for, i.e. only participants with high impact chronic pain at baseline and chronic pain (no matter what impact) at follow-up will be included in the sample.

SI-Figure 4

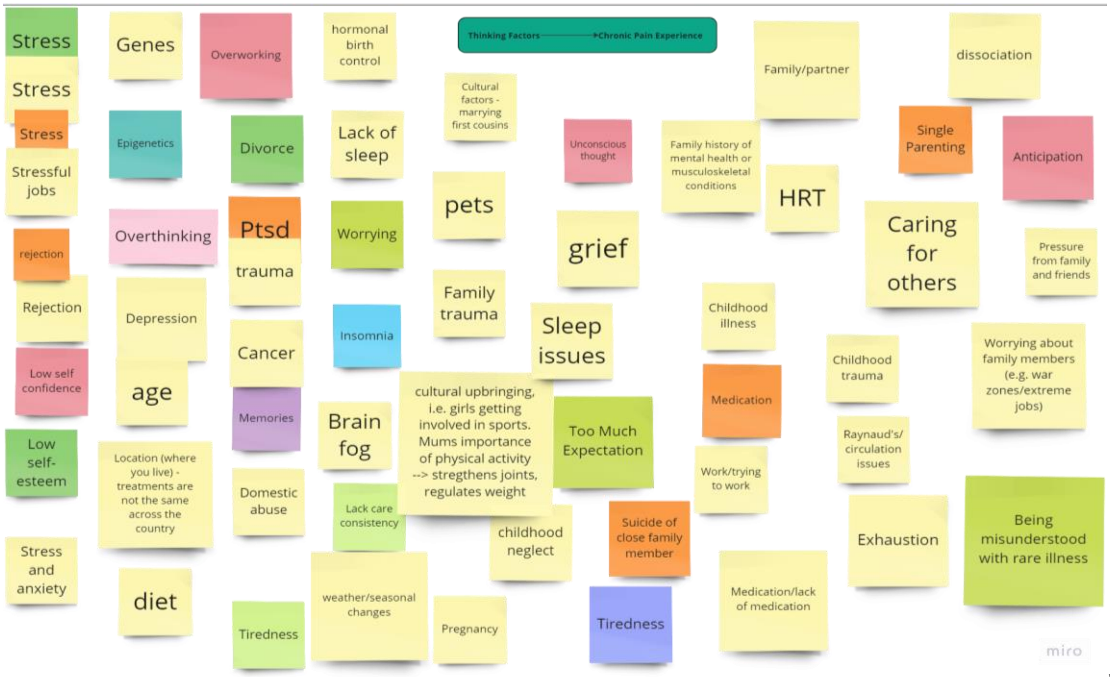

SI-

Figure 5. Results of brainstorming with individuals with lived experience of chronic pain on the common causes of 'thinking factors' and 'chronic pain experience'.

SI-Table 1

Researcher	Academic background	Expertise
Geert Crombez (GC)	Professor of Health Psychology<br>PhD in Psychology<br>MSc in Clinical Psychology	Psychology of pain, distress & disability, behavioural change, lifestyle, self-report measurement, digital interventions
Annick De Paepe (ADP)	PhD in Psychology<br>MSc in Theoretical and Experimental Psychology<br>MSc in Statistical Data-analysis	Psychology of pain, cognition, behaviour change, causal inference
Christopher Eccleston (CE)	Professor of Medical Psychology<br>PhD in Psychology<br>BSc Psychology<br>CPsychol (Practitioner Psychologist)	Pain, paediatrics, evidence-based medicine, medical psychology
Emma Fisher (EF)	PhD in Psychology<br>BSc Psychology	Pain, paediatrics, risk factors of pain transitions, evidence-based medicine.

Anna Gibby (AG)	MSc Health Psychology<br>BSc Psychology	Chronic Pain, Pain Management in Paediatric Major Trauma, Post-operative Pain
Edmund Keogh (EK)	Professor of Psychology<br>PhD in Psychology<br>MSc Occupational Psychology<br>BSc in Psychology	Psychology of pain, cognitive processes, sex/gender
Matthew Nunes (MN)	Professor of Statistics<br>PhD in Statistics<br>MMath in Mathematics	Statistical modelling, longitudinal data-analysis, computational aspects of statistical inference for large datasets
Laura Oporto Lisboa (LOL)	PhD in Statistical Applied Mathematics<br>MRes in Statistical Applied Mathematics<br>BEng Chemical Engineering	Statistical modelling, industrial and applied mathematics

SI-Table 1. *Affiliation and background of the researchers who took part in the DAG development workshops.*

SI-Table 2

Section and topic	Item	Reported on page No
**1: Aim**<br>Report the aim of the study	To develop a Directed Acyclic Graph (DAG) for the influence of executive functioning on the maintenance of high impact chronic pain. People with lived experience are an important source of domain knowledge to be able to identify all common causes of the exposure and the outcome.	P6
**2: Methods**<br>Provide a clear description of the methods used for PPI in the study	7 individuals with lived experience (ILE) of chronic pain participated in a 2-hour online workshop. During the workshop the research question and the aim of the research was introduced in non-technical terms. After this	P5-6

	ILE first thought individually about the factors that influence both their thinking ability as well as their pain ('Brainsorm phase'). Then a group discussion evolved in which we asked for clarification for several of the factors ('Refinement phase'). Finally, ILE were asked to share the three most important factors the influenced both their thinking ability and their pain.	
**3: Study results**<br>Outcomes – Report the results of PPI in the study, including both positive and negative outcomes	The IlE group generated 60 factors. The most important factors according to them were lack of care consistency (N=1), trauma (N=2), invisible illness (N=1), weather (N=2), stress, pacing (N=2), mental health (N=1), sleep (N=3), being validated (N=1), brain fog (N=1), clarity (N=1), expectation (N=1), dissociation (N=1) and energy (N=1).	P12-13<br>SI-Table 6
**4: Discussion and conclusions**<br>Outcomes – Comment on the extent to which PPI influenced the study overall. Describe positive and negative effects	There was considerable overlap between the factors mentioned by the ILE group and the ones that came up in the researcher group and the literature. Nevertheless, several factors were added to the final DAG based on the discussions in the ILE group. We decided to add 'lack of care consistency' and 'pacing' as mediators, and 'medication side-	P16-17<br>SI-Table 8

	effects' and 'trauma later in life' as common causes. Factors that were suggested by the ILE panel that were not included in the final DAG were contextual factors (e.g. treatment availability, the weather), social factors (e.g. the role of pets, stigma, social support), cognitive factors (e.g. memory, brain fog) and genetic factors.	
**5: Reflections/critical perspective** Critical perspective – Comment critically on the study, reflecting on the things that went well and those that did not, so others can learn from this experience	It was very useful to hear the reflections of the ILE group and several factors were added based on the discussions in this group. Moreover, when asked what they thought about the workshop they reported to have found it interesting and helpful. A challenge was that some of the factors that the ILE group mentioned were very personal and specific. In this case we were looking for factors that would apply to most people with chronic pain. Moreover, it was a challenge for the group to think about factors that influence both thinking ability and chronic pain and not one of them. Finally, due to a lack of time we were not able to let the ILE group think about the temporal ordering of the factors that they mentioned.	P21

SI-Table 2. GRIPP2-SF reporting checklist for the involvement of the ILE group in the study.

SI-Table 3

**Arrows**	**Decision**	**Rationale**
From transient variables<br><br>→ Impact status chronic pain (T2)	Remove	Time gap between T2 and T3 too large for variables to have an effect
Perceived stigma<br><br>→ Executive function (T1)	Remove	Works through other variables, such as low mood, anxiety,…
Environmental factors<br><br>→ Executive functioning (T1)	Remove	Works through other variables, such as comorbidities, lifestyle factors,…
Genetics<br><br>→ Executive function (T1)	Remove	Works through other variables<br>Level of explanation
Fear of pain, pain anxiety,…<br>→ Executive functioning (T1)	Remove	Only goes through transient variables
Early pain experiences<br>→ Executive functioning (T1)	Remove	Works through other variables

SI-Table 3. Results of step 3 for the researcher group.

SI-Table 4

**Change**	**Rationale**
Rename current biological state as current pain condition	Other relevant biological states go in '(medical) comorbidities'
Take pain anxiety and fear of pain together	Although some authors make a distinction between pain anxiety and pain-related fear, where the former is suggested to occur in anticipation of a painful experience, while the latter is suggested to occur during the actual experience of pain (Carleton & Asmundson, 2009), the distinction is not commonly taken into account in models of chronic pain. Therefore, we here use the term pain-related fear to refer to both pain anxiety and fear of pain.
Leave out perceived stigma, role expectations and self-identity and add profession under demographics	After consulting with experts within the field of pain-related stigma, it was decided that an association between pain-related stigma, role expectations and self-identity on the one hand and executive functioning on the other hand was unlikely. We therefore decided to leave these out of the DAG. During the discussion 'profession' came up as being related to role expectations and was added under demographics.

Leave out current social context	The restrictions in social activities are part of our definition of impact of chronic pain. There is therefore some overlap between 'social context' and the dependent variable of interest.

SI-Table 4. Additional changes made when clearly defining the constructs.

SI-Table 5

	**Definition**
**Early pain experience**	Pain experienced up to 12 months (corrected age). This can include the birth experience, procedural pain (e.g., injections, heel lances), or surgery
**Prematurity status**	Baby born alive before 37 weeks of pregnancy
**Adverse life events**	Exposure to potentially traumatic experiences, such as neglect, experiencing or witnessing violence and having a family member attempt or die by suicide, before 18 years of age that could have long-lasting effects on health and well-being (National Center for Injury Prevention and Control (U.S.), 2020)
**Parental style**	The parenting behaviours, attitudes, and emotions displayed towards a child from birth
**Demographic factors**	Age, SES (including education), first language, country of origin, profession
**Trait positive affect**	The propensity of a person to experience diverse positive emotions, such as enthusiasm, feeling active, feeling strong, feeling proud,…
**Trait negative affect**	The propensity of a person to experience diverse negative emotions, such as anxiety, stress, shame, irritation, hostility,…
**Pain duration**	Time since the onset of chronic pain
**Current pain condition**	Refers to pain condition diagnosis
**Pain-related fear**	An emotional reaction in response to actual or anticipated pain experience that causes physiological, cognitive and behavioural responses to deal with the threat of pain (Adolphs, 2013). This may involve for example fear of physical activity and fear of injury.

**Worry about pain**	A chain of thoughts and images, negatively affected laden and relatively uncontrollable in response to actual or anticipated pain experience (Borkovec et al., 1983; Eccleston & Crombez, 2007)
**Mental comorbidities**	Includes only diagnosed conditions such as depression and anxiety disorders
**Medical comorbidities**	Includes only diagnosed conditions such as multiple sclerosis, cognitive decline, ADHD, autism spectrum disorder
**Chronic somatic complaints**	Includes fatigue, balance, dizziness, sleep disorders
**Treatment exposure**	Any intervention, medication, therapy or procedure that a patient undergoes with the intention to alleviate or manage their pain symptoms, alleviate discomfort, improve function and enhance overall quality of life.
**Transient variables**	Includes momentary states of sleep, fatigue, anxiety, worry, mood, medication intake, practice effects, positive affect, negative affect
**Pain intensity**	The strength or “loudness” of the pain. This is most often measured on a 10-point numerical rating scale from 0 (no pain) to 10 (most intense pain imaginable).
**Goal adjustment processes Engagement/Disengagement**<br>**Help seeking behaviour**<br>**Treatment-related behaviour**	A person with chronic pain can be viewed as an active problem solver, who despite the persistent pain, continues to search for a solution (Eccleston & Crombez, 2007). Executive functioning may influence to what extent a person is capable of flexibly adjusting his/her goals to living with pain as opposed to keep searching for new treatments to resolve the pain. This in turn may influence the maintenance as opposed to the improvement of the impact of chronic pain.
**Treatment adherence**	The consistency and accuracy with which patients follow their prescribed pain management regimen.
**Health-related behaviours (diet, fitness, smoking)**	Lifestyle variables are influenced by some of the common causes included in our DAG

	(e.g. demographic variables). Nevertheless, even within the same levels of these variables (e.g. within participants with a certain level of education) there is some variability remaining, referred to here as 'health-related behaviours'. We hypothesise that these behaviours may be influenced by executive functioning abilities.

SI-Table 5. Definitions of all the common causes and mediators included in the DAG.

SI-Table 6

**Super node**	**Individual factors mentioned by PPIE panel (number of patients mentioning the factor if more than once)[1]**	**Details given by the PPIE panel Note that not all factors were further discussed.**
**Stress**	Stress (4 times)	Stressful life events were reported as negatively impacting thinking and pain outcomes
	Stressful job	An example given relating to the role of stress
**Demographics**	Location	Location was deemed especially important in terms of 'treatment availability'.
	Age	
**Sleep quality**	Insomnia	
	Tiredness (three times)	
	Lack of sleep	
	Sleep issues	
**Medication and care**	Hormone replacement therapy (HRT)	
	Medication availability	Sometimes there is a shortage of the drug the patients need to be able to cope with

[1] Note that every participant could see the contributions of the other PPIE members. A participant may potentially have opted not to mention a factor that was already mentioned on the board. Therefore, if a factor is only mentioned once, this does not necessarily mean that only one participant endorsed this factor.

		their pain or the medication is very expensive.
	Hormonal birth control	
	Lack of care consistency	
	Treatment options	Not many treatment options available. Patients mentioned that this causes stress. Doctors do not always mention alternative treatment options (besides medication).
	Trust in doctors	Medication can have harmful side-effects. You have to trust that doctors do not give you anything that might do harm on the long term.
	Unwanted side-effects of medication<br>~ treatment adherence	Some medication can have unwanted side-effects, such as ‘influencing thoughts’. Patients mention to weight the benefits versus the side-effects and to sometimes not adhere to treatment because of the unwanted side-effects.
**Cultural factors**	Marrying first cousins	In some cultures, the marriage of first cousins is deemed acceptable. The participants highlighted how this can result in increased likelihood of poor thinking and pain conditions due to inbreeding.
	Cultural upbringing	In some cultures girls are not encouraged to move. Patients believe that this can have an important effect later in life. A similar line or reasoning was followed for gender differences.
**Adverse childhood experiences**	Childhood illness	
	Childhood neglect	
**Trauma later in life**	Family trauma	
	Suicide of a close family member	Referring to the psychological trauma associated with unexpectedly losing a sibling to suicide.
	Post Traumatic Stress Disorder (PTSD) (two times)	

	Domestic abuse	
**Social factors**	Single parenting	An important aspect of this according to the PPIE panel is not only the fact that you have to organise everything by yourself, but also the guilt of not being able to do everything as well as you would like to do it.
	Pets	Pets were considered an important source of social support. At the same time some patients mentioned that having a pet could also have negative aspects (e.g. taking the dog out for a walk, could result in more pain)
	Presence of family/partner	Family and partner networks were considered as both helpful and detrimental factors. Whilst they were a source of support and comfort, some felt they weren't able to properly fill the role of family member to them and that their family didn't understand this. This was highlighted in both spousal and parent/child relationships.
	Social pressure	
	Rejection (two times)	This referred both to patients feeling rejected by their friends or partners who do not always know how to handle the patient with their illness as well as to the patient having to reject the partner or friends on some occasions. One of the patients gave the example of not being able to support a kiss on the cheek. For this reason intimacy is also difficult. Rejection could also be felt with respect to the doctor. A patient mentioned being labelled as 'difficult' by her doctor, because his treatment plan did not work for her.
	Worrying about family members	
	Divorce	Related to stress, one participant noted that this causes deterioration in both cognition and pain.
	Being misunderstood with a rare illness/invisible illness	One of the patients has a rare condition and feels like doctors "don't know what to make out of it". According to this patient, there is too much focus on common conditions and

	~ lack of validation	she testifies that she often feels misunderstood.
**Cognitive factors**	Brain fog	
	Unconscious thought	
	Dissociation	One of the patients described this as feeling like you are ‘out of your body’, looking down on yourself. Feeling like the world is not real. Also pain and emotions are less intense. Another patient recognised this feeling. Patients agreed that this was not a good coping strategy.
	Memories	
	Too much expectation of self	This could be both the expectations of the patient himself/herself and the expectations of other people. The former refers to the fact that the patient may still expect to be the same as before the chronic illness, but the body doesn’t live up to that expectation anymore. This results in more pain later on (‘you pay for it later’). The latter refers to the fact that others see that you sometimes do more than you actually can handle and then come to expect that you can always do those things. Stigma was also mentioned in this context.
	Worrying/anticipation	Thinking about all the things you have to do causes stress to the body. Some patients also mentioned the inability to ‘switch off’.
	Overthinking	
	Pacing	Patient explained that they tailor activities in the long run to be able to cope with their symptoms (e.g. they only work a limited amount of hours a day). Planning out all of their activities requires a lot of cognitive resources and they feel like this could potentially lead to more difficulties to keep resources for other things. As opposed to pacing also ‘frontloading’ was mentioned. This means that some patients do as much as they can in the morning when they are feeling at their best.
	Anxiety	

**Mental health factors**	Depression	
	Grief	
	Low self-esteem	
	Low self-confidence	
**Lifestyle factors**	Diet	
	Exercise	Physical activity was deemed an important factor.
**Work**	Work/trying to work	
	Overworking	Participants reported working 'too hard' when they were pain free in order to get things done. This resulted in future pain and cognitive burnout. E.g. 'I tried to get everything done that one day as I knew the next day I wouldn't be able to do anything'.
**Medical comorbidities**	Cancer	
	Raynaud's/circulation issues	A comorbidity that was reported to negatively impact pain and cognition
	Pregnancy	
**Genetics**	Genes	This was considered a strong factor by the PPIE panel. They referred to 'faulty family genes' and 'family history of mental health or musculoskeletal conditions'.
	Epigenetics	
**Environment**	weather/seasonal changes	Multiple participants recalled that their pain and ability to think is worse in colder weather

SI-Table 6. Overview of all common causes mentioned and discussed by the PPIE panel.

SI-Table 7

**Study**	Attal et al. (2014)	Vila et al. (2020)	(Giusti et al., 2020)
**Objective(s)**	Investigating the association between executive function, visual memory and attention and the development of chronic pain, its severity and neuropathic symptoms 6 months and 12 months after surgery.	Investigating the association between cognitive flexibility and persistent post-surgical pain at 6 months follow-up.	Evaluating the role of state and trait psychological variables and executive functions on pain after orthopaedic surgery, taking into account both its intensity and its components (sensory, affective and evaluative).
**Design**	Prospective longitudinal study	Prospective longitudinal study	Prospective longitudinal study
**Exposure(s) related to executive functioning**	Neuropsychological tests (Trail-Making test (TMT) A and B, Rey-Osterrieth Compex Figure copy and immediate recall) that assess cognitive flexibility, visuospatial memory and visual memory.	Trail Making test (TMT) A and B<br>Colour-Word Matching Stroop test	Trail Making Test (TMT) A and B to assess visual attention (TMT-A) and cognitive flexibility (TMT-B)
**Outcome(s)**	Development and severity of chronic pain at 6 months and 12 months	Development and severity of chronic pain at 6 months	Pain intensity and pain components (sensory, affective and evaluative) at 3 months follow-up
**N of subjects**	189 patients who underwent knee arthroplasty (N = 89) or breast surgery (N = 100)<br>181 assessed at 6 months follow-up<br>165 assessed at 12 months follow-up	300 patients who underwent knee arthroplasty (N = 150) or non-cardiac chest surgery (N = 150)<br>198 at 6 months follow-up	167 patients who underwent orthopaedic surgery<br>142 post-surgical<br>104 at 3 months follow-up

DAG(s)

DAG 1

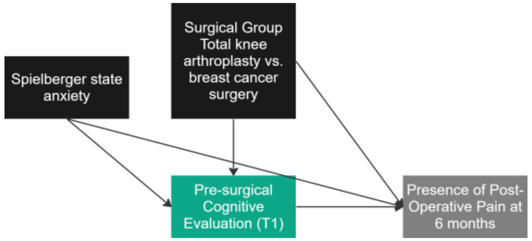

DAG 2

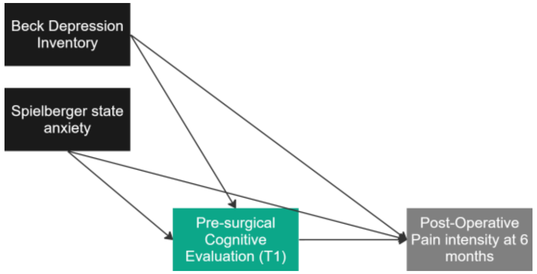

DAG 3

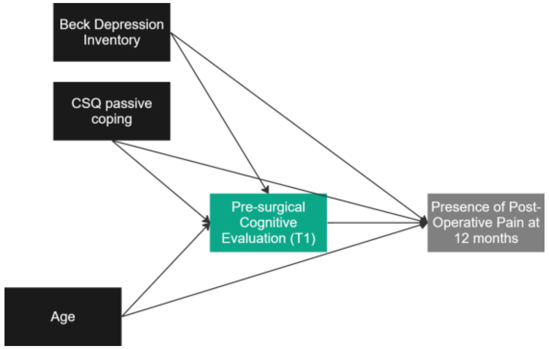

DAG 4

DAG 1

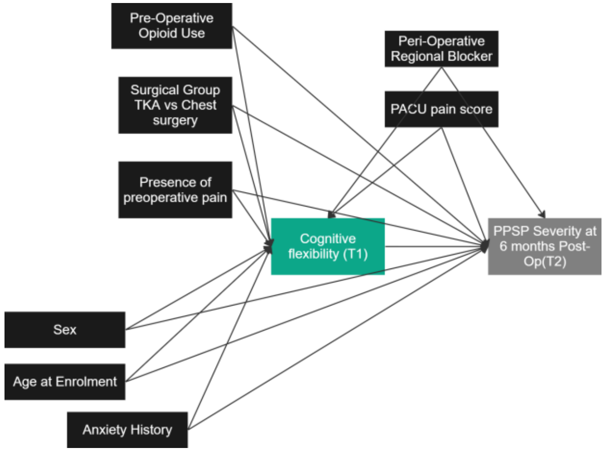

DAG 2

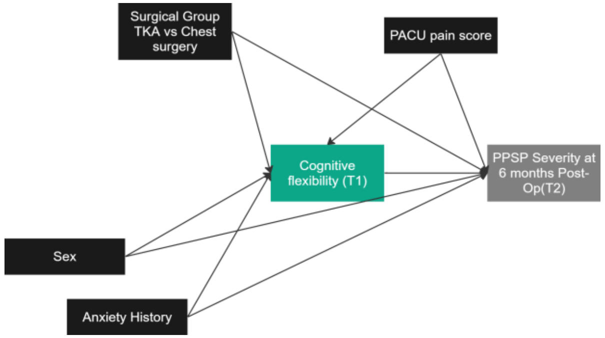

DAG 1

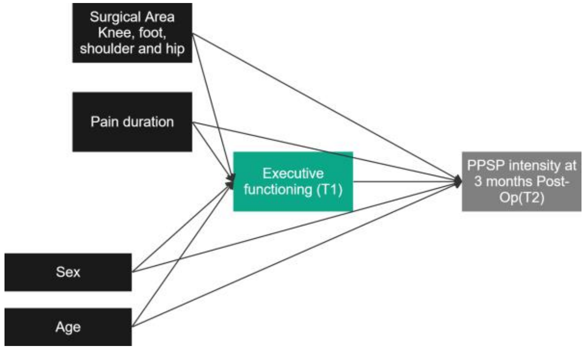

DAG 2

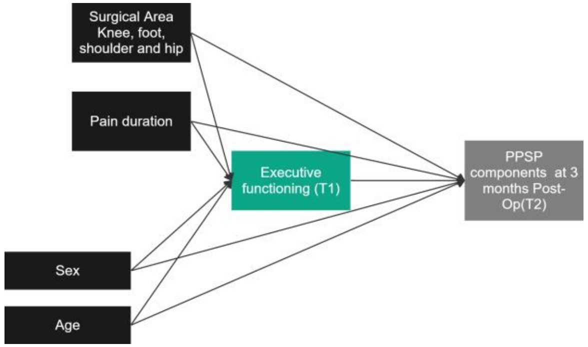

	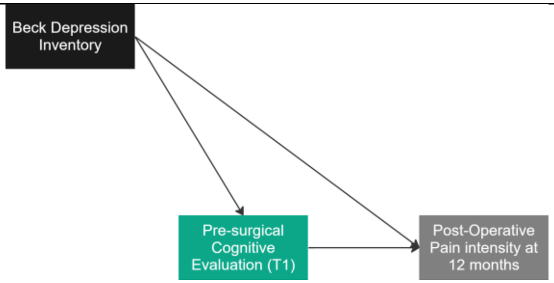		
**Results**	The presence of clinical meaningful pain (pain intensity ≥ 3) and pain intensity at 6 and 12 months was predicted by poorer cognitive performance in the Trial Making Test B, Rey-Osterrieth Complex Figure copy and recall.	Persistent Post-surgical pain incidence 6 months after surgery was not predicted by preoperative cognitive flexibility test performance. Poor performance on the colour-word matching Stroop test was associated with more severe persistent post-surgical pain.	Pain intensity at follow-up was predicted by visual attention as measured by the TMT-A.<br>The sensory component of pain were predicted by the TMT-A. The affective, evaluative and mixed components of pain were not predicted by TMT-A nor TMT-B.

**Study**	Giusti et al. (2022)	Ng & Hartanto (2022)
**Objective(s)**	Identifying biopsychosocial factors associated with acute postsurgical trajectories and with pain intensity and interference after 1, 3 and 12 months.	Investigating the predictive effect of executive function on the development of chronic pain 9 years later.
**Design**	Prospective longitudinal study	Prospective longitudinal study
**Exposure(s) related to executive functioning**	Neuropsychological assessment to evaluate executive functions:<br>Stroop Color-Word Interference Test (SCWI) to assess inability to inhibit cognitive interference<br>Attentional Matrices (AMs) to assess visual attention<br>Trail Making Test (TMT) A and B to assess attentional abilities and visual scanning and visual scanning, working memory and inhibition of habitual responses respectively.<br>TMT-B minus TMT-A was calculated to assess cognitive flexibility.	Brief Test of Adult Cognition by Telephone (BTACT) assessing working memory, inhibitory control and task switching.
**Outcome(s)**	Pain intensity and interference at 1 month, 3 months and 1 year follow-up	Presence of chronic pain and the degree of pain interference at 9 years follow-up
**N of subjects**	210 patient who underwent arthroplasty<br>186 at 1 month follow-up<br>184 at 3 months follow-up<br>165 at 1 year follow-up	1553 midlife adults<br>406 participants with chronic pain at follow-up – 399 participants were included for pain interference analysis (missing data for 7 participants)

<table>
<tr>
<td>DAG(s)</td>
<td>
DAG 1

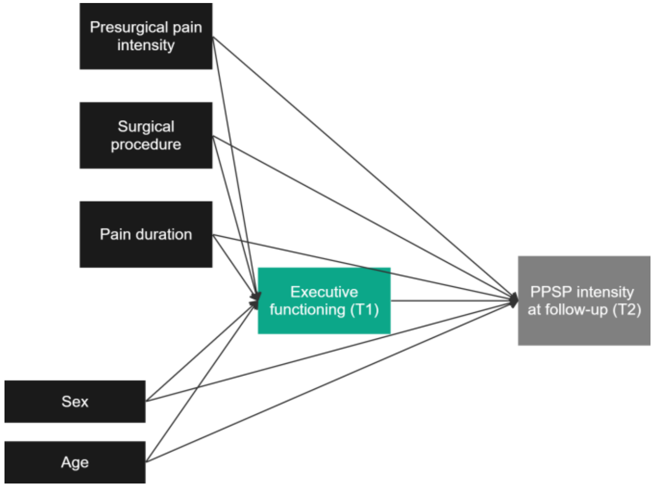

DAG 2

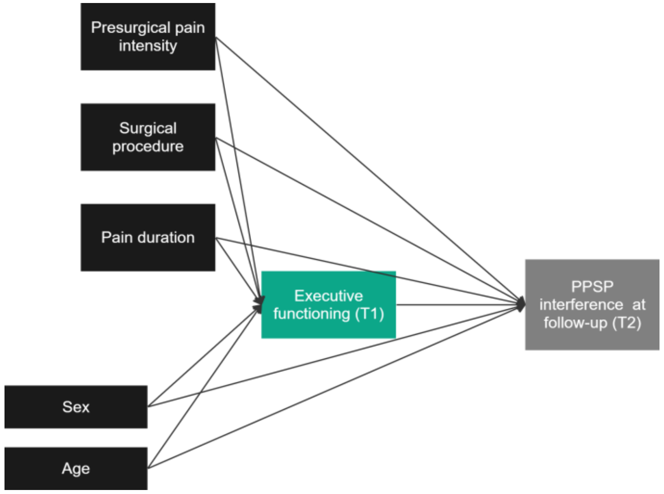

Note. The same confounders were taken into account for each follow-up period (1 month, 3 months and 1 year)
</td>
<td>
DAG 1

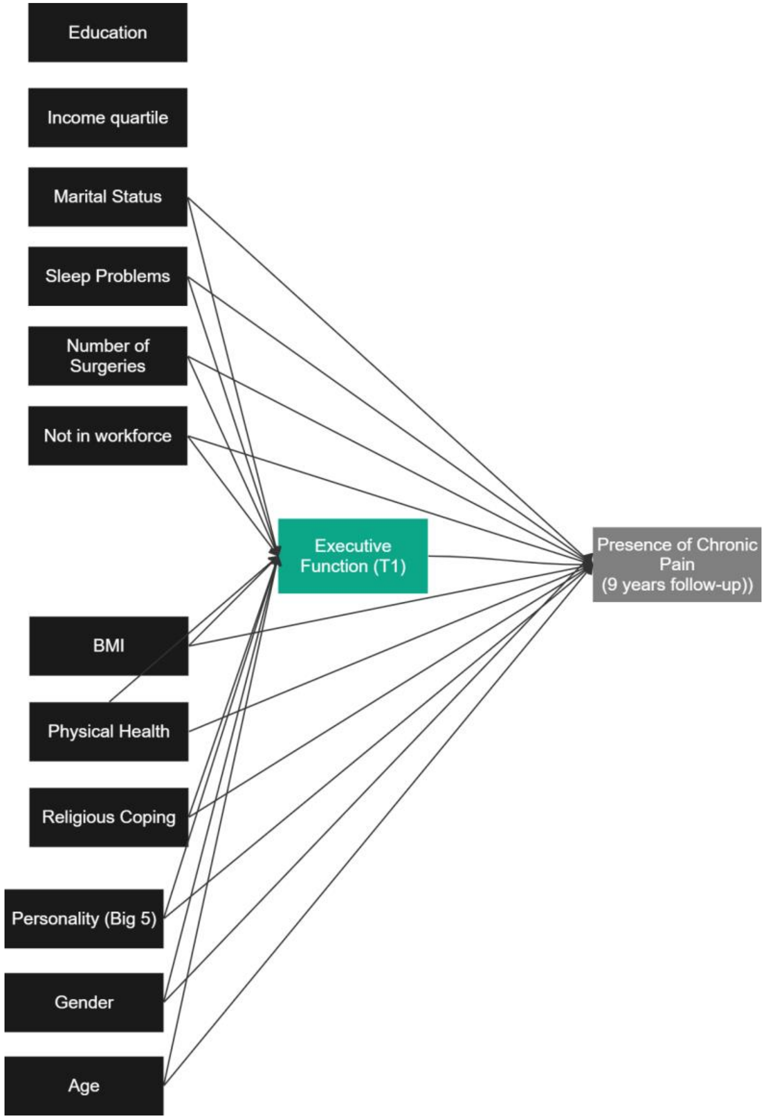

</td>
</tr>
</table>

DAG 2

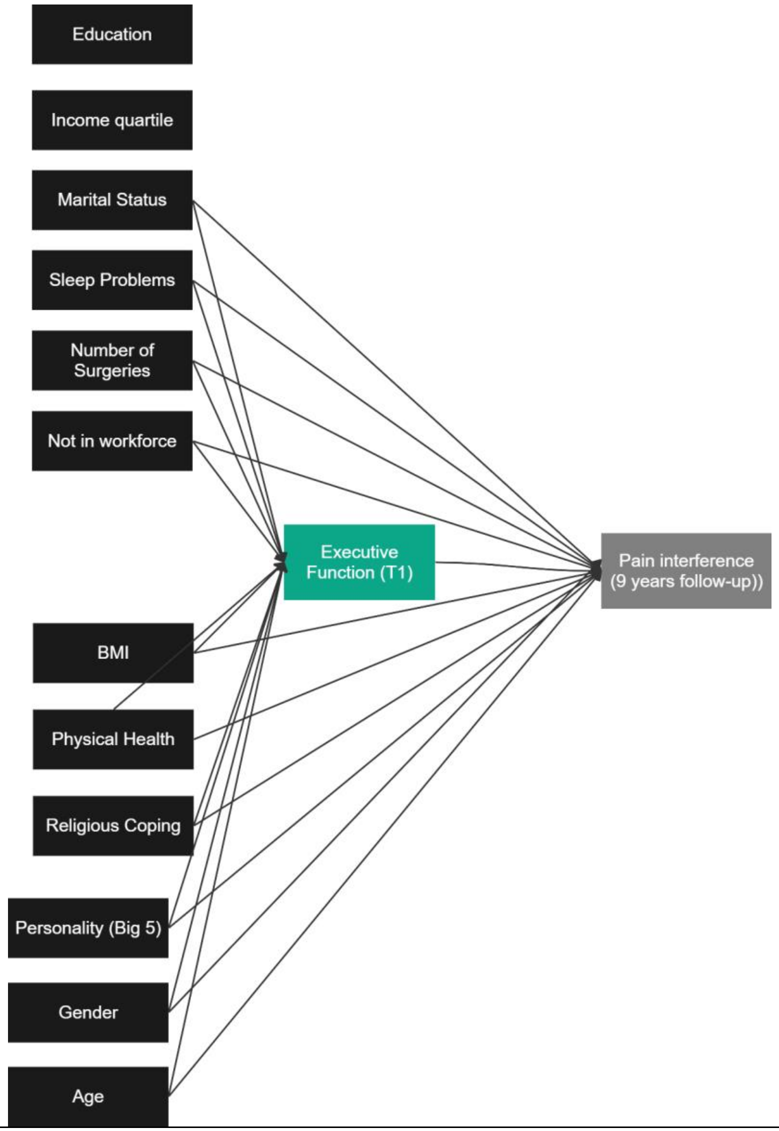

Education
Income quartile
Marital Status
Sleep Problems
Number of Surgeries
Not in workforce
Executive Function (T1)
Pain interference (9 years follow-up))
BMI
Physical Health
Religious Coping
Personality (Big 5)
Gender
Age

**Results**	Cognitive flexibility as measured by the TMT-B was associated with pain interference at 1 month follow-up. None of the other associations were significant.	Lower baseline executive function was associated with a significant likelihood of developing chronic pain 9 years later. Executive functioning failed to robustly predict the etiology of chronic pain interference 9 years later.

SI-Table 7. Overview of papers identified in the literature with their respective study objectives, design, exposures, outcomes, sample size and results. None of the papers reported a DAG and all the papers focused on 'associations' or 'predictions'. DAGs were constructed based on the statistical model reported in the papers, assuming that variables that were included as covariates in the models were considered confounders.

SI – Table 8

**Common cause**	**Source for adding common cause**	**Decision**	**Rationale**
Demographic factors – marital status	Literature/ILE	Do not include	Marital status is not important in itself, but whether or not people feel supported by other people in their environment. We consider it very unlikely that social support will have an influence on executive functioning (see further).
Demographic factors – income quartile, employment and profession	Literature	Include under SES	These factors are more precise indicators of SES and should be included. Also housing can be added there.
Demographic factors – sex	Literature	Include	Important relationship with the dependent variable of interest. It may be important to include this as a moderator or to perform sensitivity analyses (are the results the same for males and for females?).
Treatment availability	ILE	Do not include	Contextual variable that is mostly covered by treatment exposure. Include in definition of treatment exposure that treatment availability is a necessary condition. The same applies for treatment options. It is important to take contextual aspects into account when describing factors.
Lack of care consistency	ILE	Include as mediator	Executive functioning could influence care consistency and that in turn could influence chronic pain impact.
Medication side effects	ILE	Include	This most likely has an influence on the impact of chronic pain via treatment adherence.
Low self-esteem	ILE	Do not include	This will either be part of negative affect or will influence

			executive functioning via negative affect.
Personality	Literature	Do not include	Negative affectivity is the most important trait and there is considerable overlap with 'fear of pain'. If 'fear of pain' is not available, then personality may be a valuable alternative. Neuroticism and optimism may be used as proxies of trait negative and positive affect respectively.
Passive coping	Literature	Do not include	Overlap with pain-related worry.
Religious coping	Literature	Do not include	No relationship with executive functioning.
Trauma later in life	ILE	Include	There was consensus that this is an important factor to include.
Married first cousins	ILE	Do not include	The influence of this factors on executive functioning and impact of chronic pain will most likely be due to genetic factors. We decided to leave genetic factors out (see SI-Table 1).
Cultural upbringing	ILE	Do not include	This will have an influence via country of origin and first language, which are both already included in the DAG.
Social factors (stigma, social support, pets)	ILE	Do not include	We also considered this factor in the expert DAG, but was left out in the end after consulting other experts. We feel that social support and stigma will not influence executive functioning, other than through negative affect. These factors are however important for the impact of chronic pain.
Weather	ILE	Do not include	Since our research question concerns a longitudinal study where the independent variable of interest (executive

			functioning) is measured six months to 5 years before the outcome of interest (chronic pain impact) only relatively stable differences in weather may be of importance. Given the context of UK Biobank this variable is therefore not of importance.
Pacing	ILE	Include as mediator	The ability to do pacing will most likely be influenced by executive functioning abilities as opposed to the other way around.
Brain fog	ILE	Do not include	This is a very broad term, that includes executive functioning. This is partly included in transient variables included in our DAG.
Memory	ILE	Do not include	This is also part of executive functioning. Executive functioning is broader than what is measured by the trail making task. As such by including this, we may control for part of the effect that we are interested in.

SI-Table 8. Reasoning for including or not including variables suggested by the ILE and literature in the final DAG.

References

Adolphs, R. (2013). The biology of fear. In *Current Biology* (Vol. 23, Issue 2). https://doi.org/10.1016/j.cub.2012.11.055

Attal, N., Masselin-Dubois, A., Martinez, V., Jayr, C., Albi, A., Fermanian, J., Bouhassira, D., & Baudic, S. (2014). Does cognitive functioning predict chronic pain? Results from a prospective surgical cohort. *Brain*, *137*(3), 904–917. https://doi.org/10.1093/brain/awt354

Borkovec, T. D., Robinson, woon, Pruzinsky, T., & Depree, J. A. (1983). *PRELIMINARY EXPLORATION OF WORRY: SOME CHARACTERISTICS AND PROCESSES* (Vol. 21).

National Center for Injury Prevention and Control (U.S.). (2020). *National Center for Injury Prevention and Control adverse childhood experiences prevention strategy FY2021-FY2024*. https://stacks.cdc.gov/view/cdc/108183

Eccleston, C., & Crombez, G. (2007). Worry and chronic pain: A misdirected problem solving model. In *Pain* (Vol. 132, Issue 3, pp. 233–236). https://doi.org/10.1016/j.pain.2007.09.014

Giusti, E. M., Lacerenza, M., Gabrielli, S., Manzoni, G. M., Manna, C., D'Amario, F., Marcacci, M., & Castelnuovo, G. (2022). Psychological factors and trajectories of post-surgical pain: A longitudinal prospective study. *Pain Practice*, *22*(2), 159–170. https://doi.org/10.1111/papr.13074

Giusti, E. M., Manna, C., Varallo, G., Cattivelli, R., Manzoni, G. M., Gabrielli, S., D'amario, F., Lacerenza, M., & Castelnuovo, G. (2020). The predictive role of executive functions and psychological factors on chronic pain after orthopaedic surgery: A longitudinal cohort study. *Brain Sciences*, *10*(10), 1–10. https://doi.org/10.3390/brainsci10100685

Ng, W. Q., & Hartanto, A. (2022). The effect of executive function on the development of chronic pain: A prospective longitudinal study. *Social Science and Medicine*, *314*. https://doi.org/10.1016/j.socscimed.2022.115478

Vila, M. R., Todorovic, M. S., Tang, C., Fisher, M., Steinberg, A., Field, B., Bottros, M. M., Avidan, M. S., & Haroutounian, S. (2020). Cognitive flexibility and persistent post-surgical pain: the FLEXCAPP prospective observational study. *British Journal of Anaesthesia*, *124*(5), 614–622. https://doi.org/10.1016/j.bja.2020.02.002
